# Training and Racing Behavior of Recreational Runners by Race Distance—Results From the NURMI Study (Step 1)

**DOI:** 10.3389/fphys.2021.620404

**Published:** 2021-02-04

**Authors:** Beat Knechtle, Derrick R. Tanous, Gerold Wirnitzer, Claus Leitzmann, Thomas Rosemann, Volker Scheer, Katharina Wirnitzer

**Affiliations:** ^1^Medbase St. Gallen Am Vadianplatz, St. Gallen, Switzerland; ^2^Department of Sport Science, University of Innsbruck, Innsbruck, Austria; ^3^AdventureV & change2V, Stans, Austria; ^4^Institute of Nutrition, University of Gießen, Gießen, Germany; ^5^Institute of Primary Care, University of Zurich, Zurich, Switzerland; ^6^Ultra Sports Science Foundation, Pierre-Bénite, France; ^7^Department of Subject Didactics and Educational Research and Development, University College of Teacher Education Tyrol, Innsbruck, Austria; ^8^Life and Health Science Cluster Tirol, Subcluster Health/Medicine/Psychology, Innsbruck, Austria; ^9^Research Center Medical Humanities, Leopold-Franzens University of Innsbruck, Innsbruck, Austria

**Keywords:** running, marathon, half-marathon, training, race

## Abstract

The present study investigated pre-race preparation of a large sample of recreational runners competing in different race distances (e.g., shorter than half-marathon, half-marathon, marathon and ultra-marathon). An online questionnaire was used and a total of 3,835 participants completed the survey. Of those participants, 2,864 (75%) met the inclusion criteria and 1,628 (57%) women and 1,236 (43%) men remained after data clearance. Participants were categorized according to race distance in half-marathon (HM), and marathon/ultra-marathon (M/UM). Marathon and ultra-marathon data were pooled since the marathon distance is included in an ultra-marathon. The most important findings were (i) marathon and ultra-marathon runners were more likely to seek advice from a professional trainer, and (ii) spring was most commonly reported across all subgroups as the planned season for racing, (iii) training volume increased with increasing race distance, and (iv) male runners invested more time in training compared to female runners. In summary, runners competing in different race distances prepare differently for their planned race.

**Clinical Trial Registration:** www.ClinicalTrials.gov, identifier ISRCTN73074080. Retrospectively registered 12th June 2015.

## Introduction

Road based running races are held over different distances from 5 km to ultra-marathon distances of 100 km and longer (Deaner and Mitchell, 2011; Deaner et al., 2016; Knechtle et al., 2018b). In recent years, the number of successful participants of running events has increased, such as large city marathons (Knechtle et al., 2018a; Vitti et al., 2020) and ultra-marathons (Knechtle and Nikolaidis, 2017). Different studies have investigated the pre-race preparation for different race distances such as half-marathon (Damsted et al., 2019), marathon (Gordon et al., 2017) and ultra-marathon (Tokudome et al., 2004), but no study has investigated different distances, from shorter than half-marathon, to marathon and ultra-marathon in one analysis.

Regarding pre-race preparation, different/various areas of intervention like training (McKelvie et al., 1985), personality (Nikolaidis et al., 2018), motivation (Nikolaidis et al., 2019), environmental conditions (Martin, 2007), and nutrition (Burke et al., 2007) have to be considered. Moreover, when it comes down to race performance/regarding race performance, pre-race preparation is a crucial factor/is key with its different aspects such as previous experience (Bale et al., 1986; Knechtle et al., 2011a; Salinero et al., 2017) training intensity (i.e., running speed during training) (Bale et al., 1985; McKelvie et al., 1985; Knechtle et al., 2011b; Rüst et al., 2011; Hamstra-Wright et al., 2013), training volume (i.e., running kilometers, running hours) (Bale et al., 1985, 1986; Yeung et al., 2001; Hamstra-Wright et al., 2013; Salinero et al., 2017; Fokkema et al., 2020), and number of training sessions (Bale et al., 1985, 1986; Hamstra-Wright et al., 2013).

A further important aspect regarding pre-race preparation and competing is also the selection of a specific race and season. When we consider some of the largest marathons in the world, the “Boston Marathon” is held in spring (third Monday in/end of April) (Maffetone et al., 2017) whereas other large city marathons such as the “New York City Marathon” (Gasparetto and Nesseler, 2020) and the “Berlin Marathon” (Muñoz-Pérez et al., 2020) are held in autumn where “Berlin Marathon” is held in middle or end of September and “New York City Marathon” on the first Sunday of November. The timing of competing in a marathon might be of relevance for the preparation of an athlete.

Therefore, the present study is the first to investigate training, preparing and racing behavior of recreational runners performed on a large sample of recreational runners competing in different race distances (e.g., shorter than half-marathon, half-marathon, marathon, and ultra-marathon). Based on existing findings, we hypothesized that runners of different race distances would prepare differently for their specific race distance.

## Materials and Methods

### Study Protocol and Ethics Approval

The ethics board of St. Gallen, Switzerland approved the protocol of the NURMI (Nutrition and Running High Mileage) Study (Wirnitzer et al., 2016) on May 6, 2015 (EKSG 14/145). The trial registration number is ISRCTN73074080.

### Participants

Runners were contacted and recruited mainly via social media, websites of the organizers of marathon events, online running communities, personal contacts, and email-lists of runners’ magazines as well as health magazines, including nutrition and lifestyle, trade fairs on sports, and plant-based nutrition and lifestyle. Although it was not our target (main regions intended to be addressed were European countries with German-speaking countries, such as Austria, Germany and Switzerland as core regions), the online-survey was spread across the globe too, by disseminating the information of this study within the international and global runners’ community. Therefore, this additional sample of 75 highly motivated runners from non-European nations provided valuable data by giving numerous accurate and useful answers. In order to avoid an irreversible loss of these valuable data sets, those who met all inclusion criteria were enrolled in the study in order to create a bigger sample size, and thus increase representativity of data provided with the current results. The demographics and characteristics of the participants are presented in Table 1.

**TABLE 1:** Demographics and characteristics of participants displayed by race distance subgroup.

		Total	<21 km	HM	M/UM
Number of participants		2,864 (100%)	622 (22%)	1,032 (36%)	1,210 (42%)
**Sex**					
	Female	1,628 (57%)	468 (75%)	652 (63%)	508 (42%)
	Male	1,236 (43%)	154 (25%)	380 (37%)	702 (58%)
Age (years)		37 (IQR 17)	32 (IQR 16)	34 (IQR 16)	41 (IQR 15)
Body weight (kg)		66 (IQR 16)	63 (IQR 15)	65 (IQR 15)	68 (IQR 16)
Height (m)		1.73 (IQR 0.13)	1.70 (IQR 0.12)	1.72 (IQR 0.12)	1.75 (IQR 0.13)
**BMI_CALC_ (kg/m^2^)**					
	18.50–24.99	2,394 (83%)	501 (80%)	866 (84%)	1,027 (85%)
	<18.50	138 (5%)	42 (7%)	46 (4%)	50 (4%)
	>24.99	332 (12%)	79 (13%)	120 (12%)	133 (11%)
**Race distance/s completed**					
	<21 km	1,099 (38%)	622(100%)	281 (27%)	196 (16%)
	Half-marathon	1,979 (69%)	/	1,032 (100%)	947 (78%)
	Marathon	1,148 (40%)	/	/	1148 (95%)
	Ultra-marathon	336 (12%)	/	/	336 (28%)
**Nationality**					
	Europe	2,789 (97%)	607 (98%)	1,002 (97%)	1,180 (98%)
	America	70 (2%)	14 (2%)	29 (3%)	27 (2%)
	Asia (=India)	4 (<1%)	1 (<1%)	1 (< 1%)	2 (<1%)
	Other (n.a.)	1 (<1%)	/	/	1 (<1%)

*Results are presented as total numbers, percentage (%), and median (IQR). <21 km–less than half marathon; HM, half marathon; M/UM, marathon/ultra-marathon. BMI_*CALC*_, Body Mass Index calculated and categorized following the [World Health Organization (WHO)].*

### Procedures

#### Experimental Approach

The NURMI Study was conducted in three steps following a cross-sectional design. Step 1 (preliminary study) aimed to determine “Who is running?” meaning the prevalence of runners who are at the start of running events considering race distance, running training and race preparation etc. The participants completed a short online-survey within the NURMI Study Step 1, provided in German and English, which was available on www.nurmi-study.com from October 1st, 2014, until December 31st, 2015. The survey started with a written description of the procedure and participants gave their informed consent to take part in the study. Afterward, they completed the questionnaire, concerning demographic characteristics, current adherence to a specific kind of diet, and distance/s active in running (training, races). Particularly, it consisted of seven parts with a total of 38 questions about the individual (1), running races (2) and running training (3), planned running races for the current and subsequent season (4), and miscellaneous (5). In order to identify conflicting data and to obtain the most reliable data possible, control questions were included.

For a successful participation in the study, the following four inclusion criteria were required: (1) written informed consent, (2) at least 18 years of age, (3) questionnaire Step 1 completed retrospectively to a race, and (4) completion of a running event in the past two years and still active in running (all distances, all levels). Those who met all inclusion criteria were enrolled into the data analysis.

Participants were categorized according to race distance (Table 1): half-marathon (HM), and marathon/ultra-marathon (M/UM: data were pooled since the marathon distance is included in an ultra-marathon). The shortest ultra-marathon-distance reported was 50 km and the longest distance was 160 km. In addition, a total of 622 highly motivated runners provided accurate and useful answers with plenty of high-quality data. However, they had not successfully participated in either a HM or M, but had participated in a race distance shorter than HM instead. In order to avoid an irreversible loss of these valuable data sets, those who met all inclusion criteria, but reported races shorter than HM (<21 km) race as their running event, were kept as an additional race distance subgroup.

According to the (World Health Organization [WHO], 2018a,b) the goal for individuals should be to maintain a BMI in the range 18.5–24.9 kg/m^2^ (BMI_NORM_) in order to achieve optimum health. A BMI of 25.0–29.9 kg/m^2^ points to an increased risk of co-morbidities, and moderate to severe risk of co-morbidities for a BMI >30 kg/m^2^ (World Health Organization [WHO], 2018a,b). Therefore, the calculated Body Mass Index (BMI_CALC_) was classified into three categories of the body weight-to-height ratio (kg/m^2^): ≤18.49 < BMI_NORM_: 18.50–24.99 kg/m^2^ ≥ 25. This was completed because the BMI of active runners could be below BMI_NORM_ (Cheuvront et al., 2005; Thibault et al., 2010; Manore, 2015), and participants with a BMI <30 kg/m^2^ were included because some people with a higher BMI might start running in order to achie4ve and maintain a stable, healthy body weight.

#### Data Clearance

More than 7,400 participants started to fill in the online-survey. However, 48% dropped out, with a total of 3,835 runners who completed the survey. Incomplete, inconsistent and conflicting data sets were excluded from data analysis (*n* = 834). In order to control for measures of (1) running activity and (2) diet, two groups of control questions were included, each within different sections of the survey. A total of 156 participants with no statement about running training (e.g., training time) were excluded from data analysis. Moreover, in order to control for a minimal status of health linked to a minimum fitness level, and to further enhance the reliability of data sets, the BMI approach followed the (World Health Organization [WHO], 2018a,b). However, with a BMI ≥30 kg/m^2^, additional health protective and/or weight loss strategies other than running are necessary to safely reduce body weight first, to reduce body weight with no risk. Therefore, 42 participants with a BMI ≥30 kg/m^2^ were excluded from data analysis.

**FIGURE 1:**
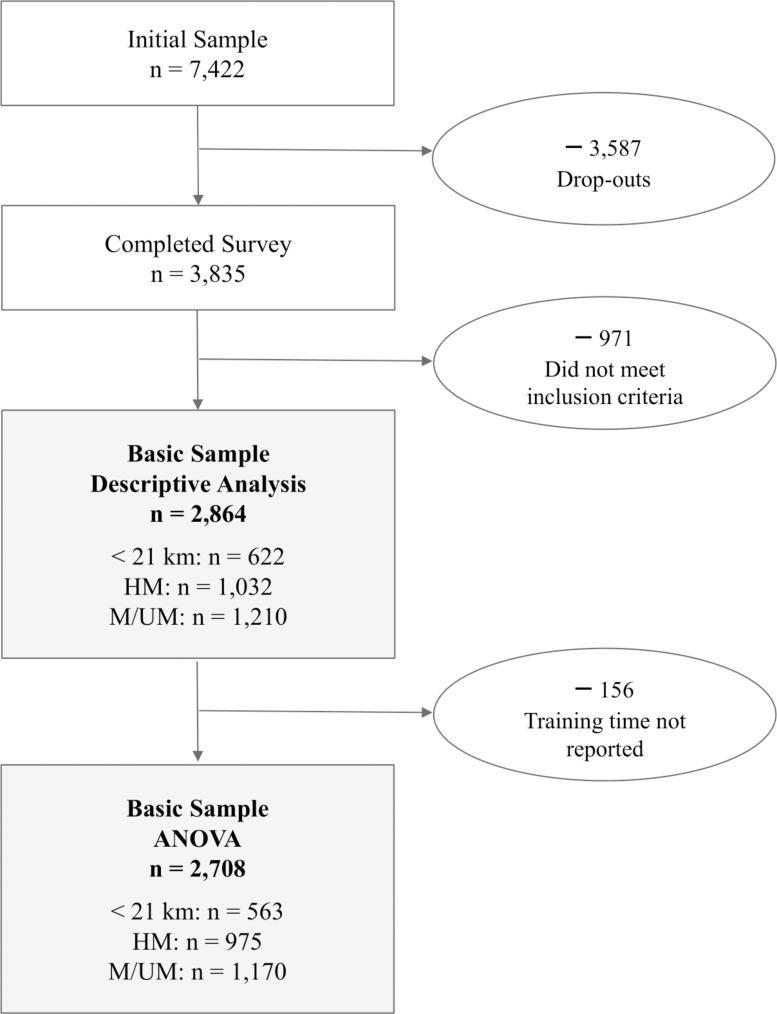
Flow of participants’ enrollment NURMI Study Step 1.

After data clearance, a total of 2,864 recreational runners (1,628 women, 1,236 men) with complete data sets were included for *descriptive* statistical analysis. Those active in running events were included in further statistical analyses. In order to control for the latter, the individual best runtimes were verified by randomly selected cases. Figure 1 shows the flow of participants’ enrollment for the NURMI Study Step 1.

#### Measures

Prevalence of (endurance) runners at the start of running events was described using an epidemiological approach performed by the following items: nationality, age, sex, body weight, height, BMI_CALC_. [World Health Organization (WHO)]; training behavior (weekly/daily time spend in running/day), period of time to prepare for the main running event; aim of taking part in a running race (performance vs. joyful/enjoyment approach); participation in running events, distance/s that have been successfully completed (<21 KM, HM, M, UM), number of specific distance/s completed, individual best time over HM and/or M distance/s;

### Statistical Analysis

The statistical software R version 3.6.2 Core Team 2019 (R Foundation for Statistical Computing, Vienna, Austria) was used to perform all statistical analyses. Exploratory analysis was performed by descriptive statistics with continuous variables summarized as median and interquartile ranges (IQRs) or mean values and standard deviation (SD), and categorical variables summarized as percentages. Significant differences between race distance subgroups and both sex and training mileage (low, medium, high, e.g., daily or per week) were calculated by using a non-parametric ANOVA to describe running habits (training, race). Factors associated with the respective variables were examined by univariate analysis using Chi-square test (χ^2^; nominal scale) for categorical variables, and Kruskal-Wallis test (ordinal and metric scale) approximated by t or F distributions using ordinary least squares, standard errors (SE) and *R*^2^ for continuous variables. In order to relate weekly training by distance in female and male runners a linear regression model was performed with differences (marginal effects) in the respective variables are displayed as effect plots [95% confidence interval (95%-CI)]. The level of statistical significance was set at *p* ≤ 0.05.

## Results

A total of 3,835 participants completed the survey. Of those participants, 2,864 (75%) met the inclusion criteria, and 1,628 (57%) women and 1,236 (43%) men remained after data clearance. The median age is 37 (IRQ 17, range: 18–74) years, with a median body weight of 66 (IRQ 16, range: 40–105) kg, a median height of 1.73 (IRQ 0.13, range: 1.34–2.40) m, and a median BMI_CALC_ of 22.0 (IRQ 3.3, range: 11.4–29.9) kg/m^2^. 2,478 (84%) NURMI runners were within the BMI_NORM_, 141 (5%) reported a BMI <18.5 kg/m^2^ and 340 (11%) reported a BMI > 24.99 kg/m^2^. The regions of origin included Europe (*n* = 2,789; 97%), America (*n* = 70; 2%) and Asia (*n* = 4; < 1%). With regard to race distances, there were 622 (22%) runners over a distance of less than half-marathon (<21 km), 1,032 (36%) half-marathoners, and 1,210 (42%) marathoners/ultra-marathoners. Demographics and characteristics of the participants are presented in Table 1.

### Racing Behavior of Recreational Runners of <21 km, HM and M/UM

Table 2 displays the participants in HM and M/UM subgroups as none of the participants included in the <21 km subgroup completed a half-marathon, marathon, or ultra-marathon race. A total of 947 half-marathons were completed by the M/UM subgroup. M/UM runners reported a faster half marathon best time (102 min) on average compared to the HM subgroup (118 min). None of the participants of the HM subgroup completed a marathon or ultra-marathon. A total of 1,148 marathons were completed by the M/UM subgroup. The best time for the M/UM running subgroup to complete a marathon was 228 min on average. A total of 336 ultra-marathons were completed by the M/UM subgroup with a best time of 751 min on average.

**TABLE 2:** Number of completed half-marathon, marathon, and ultra-marathon events.

		HM	M/UM
**Half-marathon**			
	**Completed events**		
	Total	1,032	947
	Median	2 (IQR 3, range 1–50)	6 (IQR 8, range 1–76)
	1	340 (33%)	56 (6%)
	2	248 (24%)	118 (12%)
	3–4	224 (22%)	192 (20%)
	5–7	119 (12%)	195 (21%)
	>7	99 (10%)	383 (40%)
	n.a.	2 (<1%)	3 (<1%)
	Best time	118.4 (*SD* 22.1)	101.6 (*SD* 16.3)
**Marathon**			
	**Completed events**		
	Total	0	1,148
	Median	/	3 (IQR 5, range 1–97)
	1	/	305 (27%)
	2	/	208 (18%)
	3–4	/	230 (20%)
	5–7	/	152 (13%)
	>7	/	248 (22%)
	n.a.	/	5 (<1%)
	Best time	/	227.8 (*SD* 39.9)
**Ultra-marathon**			
	**Completed events**		
	Total	0	336
	Median	/	3 (IQR 4, range 1–43)
	1	/	75 (22%)
	2	/	56 (17%)
	3–4	/	64 (19%)
	5–7	/	47 (14%)
	>7	/	40 (12%)
	n.a.	/	54 (16%)
	Best time	/	750.8 (*SD* 201.4)

*Best time displayed by HM and M/UM subgroups in minutes [mean with standard deviation (SD)]. Results are presented as total numbers, percentage (%), mean, and standard deviation (SD). HM, half-marathon; M/UM, marathon/ultra-marathon.*

### Season of Planned Race

Spring is most commonly reported across all subgroups as the planned season for racing (see statistics, Table 3). Moreover, 2015 is most commonly reported across all subgroups as the planned year for running events. All of the participants planned for one of the three events, but not all participants planned for a year or a season. The “other” category of planned event distance includes the following races: mountain run, triathlon, 12 h run, or ironman. The majority of runners included in the <21 km subgroup (*n* = 424) planned to complete a race of less than half-marathon for their next event and the remaining 165 runners planned to complete a half-marathon. The majority of subjects included in the HM subgroup (*n* = 513) planned to complete a half-marathon race for their next event, 292 HM runners planned to complete a distance of less than a half-marathon, and 202 planned to complete a marathon. The majority of subjects included in the M/UM subgroup (*n* = 587) planned to complete a marathon race for their next event, 421 M/UM runners planned to complete a distance of less than a marathon, and 180 planned to complete an ultra-marathon. The second and third event planned is most commonly reported as less than half-marathon for each subgroup.

**TABLE 3:** Planned event distance/s, year/s, and season/s displayed by race distance subgroup.

		<21 km	HM	M/UM
**Event 1: Distance, year, season**				
	<21 km	68% (424)	28% (292)	16% (188)
	HM	27% (165)	50% (513)	19% (233)
	M	4% (22)	20% (202)	49% (587)
	UM	1% (8)	2% (19)	15% (180)
	Other	< 1% (3)	<1% (6)	2% (22)
	2014	14% (68)	11% (99)	9% (103)
	2015	84% (397)	86% (761)	88% (967)
	2016	1% (7)	2% (20)	2% (24)
	Winter	7% (31)	6% (52)	7% (72)
	Spring	51% (241)	47% (414)	44% (482)
	Summer	24% (114)	21% (187)	21% (235)
	Fall	19% (88)	26% (227)	28% (309)
**Event 2: Distance, year, season**				
	<21 km	88% (548)	60% (619)	38% (465)
	HM	10% (60)	29% (299)	22% (261)
	M	1% (7)	8% (87)	26% (312)
	UM	<1% (3)	2% (20)	13% (153)
	Other	<1% (4)	<1% (7)	2% (19)
	2014	10% (28)	9% (58)	7% (62)
	2015	89% (255)	89% (571)	91% (790)
	2016	2% (5)	2% (13)	2% (18)
	Winter	8% (24)	9% (57)	10% (83)
	Spring	46% (132)	39% (252)	42% (366)
	Summer	26% (75)	29% (187)	24% (211)
	Fall	20% (59)	23% (147)	24% (212)
**Event 3: Distance, year, season**				
	<21 km	95% (592)	86% (886)	70% (845)
	HM	4% (25)	10% (104)	12% (140)
	M	<1% (2)	3% (31)	11% (139)
	UM	<1% (2)	<1% (7)	6% (76)
	Other	< 1% (1)	<1% (4)	<1% (10)
	2014	8% (8)	11% (29)	8% (37)
	2015	92% (97)	88% (232)	91% (413)
	2016	<1% (1)	1% (3)	1% (6)
	Winter	10% (11)	7% (18)	9% (39)
	Spring	42% (44)	34% (90)	38% (173)
	Summer	28% (30)	32% (85)	30% (135)
	Fall	20% (21)	27% (71)	24% (110)

*Results are presented as total numbers and percentage (%). <21 km, less than half-marathon; HM, half-marathon; M, marathon; UM, ultra-marathon; Other, mountain run, triathlon, 12-h run, or ironman.*

### Training Behavior of Recreational Runners of <21 km, HM and M/UM

Kruskal-Wallis tests (see statistics, Table 4) indicated that M/UM runners train (i) for the longest time period for running events (*p* < 0.001), (ii) at the highest weekly running frequency (*p* < 0.001), (iii) at the highest running mileage per week (*p* < 0.001), (iv) at the highest running mileage per day (*p* < 0.001), (v) at the longest running duration per week (*p* < 0.001), and (vi) at the longest running duration per day (*p* < 0.001) followed by HM, and < 21 km subgroups (i–vi). Table 5 shows M/UM runners are the most likely subgroup to train under the direction of a professional followed by HM, and <21 km subgroups (*p* = 0.012). No significant difference between subgroups and the type of professional direction were found: performance assessment (*p* = 0.126); trainer (*p* = 0.251); sports scientist (*p* = 0.511); or doctor specialized in sports medicine (*p* = 0.802).

**TABLE 4:** Training period, frequency, mileage, and duration displayed in total and by race distance subgroup.

		Total	<21 km	HM	M/UM	Test statistic
Number of participants		2,708 (100%)	563 (21%)	975 (36%)	1,170 (43%)	/
**Training time period**						
	1–2 months	27% (781)	42% (264)	30% (306)	17% (211)	H_(2)_ = 103.18 *p* < 0.001
	3–4 months	46% (1,322)	38% (234)	45% (468)	51% (620)	
	4–6 months	18% (514)	13% (83)	17% (174)	21% (257)	
	7–8 months	4% (110)	2% (15)	4% (37)	5% (58)	
	9–10 months	2% 64)	1% (9)	2% (20)	3% (35)	
	More than a year	3% (73)	3% (17)	3% (27)	2% (29)	
Weekly training frequency		3 (IQR 1; 1–14)	3 (IQR 1; 1–14)	3 (IQR 1; 1–7)	4 (IQR 2; 1–12)	H_(2)_ = 401.62 *p* < 0.001
**Training mileage**						
	Kilometers per week	43.3 (*SD* 25.4; 5.0–219.5)	27.5 (*SD* 18.1; 5.0–182.9)	36.3 (*SD* 19.2; 5.0–187.5)	56.7 (*SD* 26.4; 5.0–219.5)	*F*_(2, 2,705)_ = 554.94 *p* < 0.001
	Kilometers per day	13.1 (*SD* 7.3; 3.4–120.0)	10.8 (*SD* 6.5; 3.9–61.9)	12.3 (*SD* 7.2; 3.4–90.0)	14.8 (*SD* 7.3; 4.0–120.0)	*F*_(2, 2,705)_ = 179.26 *p* < 0.001
**Training time duration**						
	Hours per week	4.7 (*SD* 2.8; 0.6–24.0)	3.0 (*SD* 2.0; 0.6–20.0)	4.0 (*SD* 2.1; 0.6–20.5)	6.2 (*SD* 2.9; 0.6–24.0)	*F*_(2, 2,705)_ = 554.93 *p* < 0.001
	Hours per day	0.85 (*SD* 0.47; 0.22–7.75)	0.70 (*SD* 0.42; 0.25–4.00)	0.79 (*SD* 0.47; 0.22–5.81)	0.96 (*SD* 0.47; 0.26–7.75)	*F*_(2, 2,705)_ = 179.29 *p* < 0.001

*Results are presented as total numbers, percentage (%), mean, standard deviation (SD), median (IQR), and range (min-max). <21 km, less than half-marathon; HM, half-marathon; M/UM, marathon/ultra-marathon. Kruskal-Wallis test was performed to calculate H_(2)_ and F statistic.*

**TABLE 5:** Training type displayed in total and by race distance subgroup.

		Total	<21 km	HM	M/UM	Test statistic
**Training preparation**						
	Under the direction of a professional	10% (295)	8% (47)	10% (103)	12% (145)	χ^2(2)^ = 8.89 *p* = 0.012
	Alone and independently	90% (2,569)	92% (575)	90% (929)	88% (1,065)	
**Type of professional direction**						
	Performance assessment	32% (96)	21% (10)	31% (32)	37% (54)	χ^2(2)^ = 4.14 *p* = 0.126
	Trainer	92% (273)	98% (46)	92% (95)	90% (132)	χ^2(2)^ = 2.76 *p* = 0.251
	Sports scientist	12% (35)	9% (4)	15% (15)	11% (16)	χ^2(2)^ = 1.34 *p* = 0.511
	Doctor specializing in sports medicine	10% (29)	9% (4)	9% (9)	11% (16)	χ^2(2)^ = 0.44 *p* = 0.802

*Results are presented as total numbers and percentage (%). <21 km, less than half-marathon; HM, half-marathon; M/UM, marathon/ultra-marathon. Chi-squared test was performed to calculate $\chi_{\left(2\right)}^{2}$ statistic.*

### Training Behavior of Race Distance Subgroup, Sex, and Interaction

**FIGURE 2:**
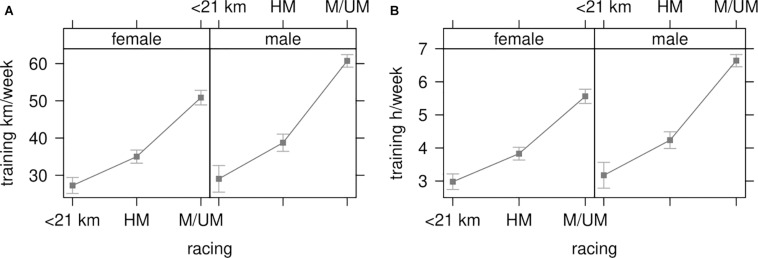
**(A)** Training interaction: race distance subgroup and sex by weekly mileage. **(B)** Training interaction: race distance subgroup and sex by weekly duration. Results are presented as mean effect sizes with bars displaying 95% confidence interval adjusted by sex and diet. <21 km, less than half marathon; HM, half marathon; M/UM, marathon/ultra-marathon; km, kilometers; h, hours.

The weekly training volume is displayed in Figure 2 (mean effect size with 95%-CI) with race distance subgroups (<21 km, HM, M/UM), sex (male, female) and their interaction. Female runners of the <21 km subgroup (*n* = 415), ran an average of 27 km (±18.13) per week (CI 27.26; 25.10–18.13) at a weekly duration of 2 h 57 min (±1 h 58 min on average (CI 2 h 58 min; 2 h 44 min–3 h 13 min). Male runners of the <21 km subgroup (*n* = 148), ran an average of 29 km (±18.13) per week (CI 29.04; 25.47–32.61) at a weekly duration of 3 h 10 min (±1 h 58 min) on average (CI 3 h 10 min; 2 h 46 min–3 h 33 min). Females runners of the HM subgroup (*n* = 619) ran an average of 34.8 km (±18.65) per week (CI 35.01; 33.26–36.77) at a weekly duration of 3 h 48 min (±2 h 2 min) on average (CI 3 h 49 min; 3 h 38 min–4 h 1 min). Male runners of the HM subgroup (*n* = 356) ran an average of 38.8 km (±19.87) per week (CI 38.75; 36.44–41.06) at a weekly duration of 4 h 14 min (±2 h 10 min) on average (CI 4 h 14 min; 3 h 58 min–4 h 29 min). Female runners of the M/UM subgroup (*n* = 485), ran an average of 50.9 km (±22.26) per week (CI 50.87; 48.89–52.84) at a weekly duration of 5 h 33 min (±2 h 25 min) on average (CI 5 h 33 min; 5 h 21 min–5 h 46 min). Male runners of the M/UM subgroup (*n* = 685), ran an average of 60.7 km (±28.23) per week (CI 60.74; 59.05–62.42) at a weekly duration of 6 h 38 min (±3 h 5 min) on average (CI 6 h 38 min; 6 h 27 min–6 h 49 min).

Training in kilometers per week (Figure 2A), with race distance subgroups and sex as between-subject factors revealed a main effect of both race distance subgroup [*F*_(2, 2,698)_ = 319.58, *p* < 0.001] and sex [*F*_(1, 2,698)_ = 46.93, *p* < 0.001]. These main effects were qualified by an interaction between race distance subgroup and sex [*F*_(2, 2,698)_ = 7.43, *p* = 0.001]. Weekly training hours (Figure 2B), with race distance subgroups and sex as between-subject factors revealed a main effect of race distance subgroup [*F*_(2, 2,698)_ = 319.60, *p* < 0.001], and sex [*F*_(1, 2,698)_ = 46.94, *p* < 0.001]. These main effects were qualified by an interaction between race distance subgroup and sex [*F*_(2, 2,698)_ = 7.43, *p* = 0.001].

## Discussion

The present study intended to investigate pre-race preparation on a large sample of recreational endurance runners competing in different race distances (e.g., shorter than half-marathon, half-marathon, marathon and ultra-marathon). The most important findings were (i) marathon and ultra-marathon runners were more likely to seek advice from a professional trainer, and (ii) spring was most commonly reported across all subgroups as the planned season for racing, (iii) training volume increased with increasing race distance, and (iv) male runners invested more time in training compared to female runners.

### Marathon and Ultra-Marathoners Sought More Often Advice From a Professional Trainer

A first important finding was that runners of longer distances (i.e., marathon and ultra-marathon) sought professional help in preparing for races more often, but not runners of shorter distances (i.e., half-marathon and shorter). It is well known that runners of longer distances, such as marathoners, rely on expert-opinion and the anecdotal advice of their peers when devising their training plans for an upcoming race (Doherty et al., 2020). However, runners of shorter distances (i.e., half-marathon) may also profit from professional advice. A current study investigating recreational half-marathoners showed that athletes supported by a qualified staff for race preparation showed better results in the dimensions of physical function and emotional role (Romaratezabala et al., 2020).

### Spring Was Most Commonly Reported Across All Subgroups as the Planned Season for Competing

A second important finding was that most runners intended to compete in their races during spring. This is an important finding since large city marathons have a seasonal distribution with two peaks, one in spring (weeks 14–17) and one in autumn (weeks 41–44) (Marc et al., 2014). Although one of the largest city marathons, the “Boston Marathon” is held in spring (Maffetone et al., 2017) other large city marathons such as the “New York City Marathon” (Gasparetto and Nesseler, 2020) and the “Berlin Marathon” (Muñoz-Pérez et al., 2020) are held in autumn. A possible explanation for these recreational runners could be that they train preferably during winter in order to compete in spring.

### Training Volume Increased With Increasing Race Distance

A third important finding was that runners of longer distances (e.g., marathon and ultra-marathon) invested more time in training (i.e., more training units, more time for training, longer trainings) compared to runners of shorter distances (e.g., half-marathon and shorter race distances). This confirms existing findings (Knechtle, 2012; Rüst et al., 2012a; Teresa Zillmann et al., 2013; Turner-McGrievy et al., 2016). It is well known that training volume increases with increasing race distance. Generally, ultra-marathoners reported more years of running compared to half-marathoners and marathoners (Turner-McGrievy et al., 2016). Half-marathoners completed fewer weekly training kilometers and fewer weekly running hours compared to the marathoners (Teresa Zillmann et al., 2013). Also, for longer distances, it has been shown that ultra-marathoners (e.g., 24-h ultra-marathoners) completed more training kilometers and more training hours than marathoners, but they run slower during training than marathoners (Knechtle, 2012; Rüst et al., 2012a).

It is well-known that training is important for a successful race finish in runners. Preparation for both a half-marathon and a marathon with a relatively high training volume and long endurance runs have been associated with faster race times (Doherty et al., 2020; Fokkema et al., 2020). There are, however, differences in training between marathoners and ultra-marathoners. It has been shown that marathoners rely more on speed in running during training, whereas 100 km ultra-marathoners rely more on volume in running training (Rüst et al., 2012b).

### Male Runners Invested More Time in Training Compared to Female Runners

A last important finding was that male runners were training more than female runners. These findings seem to differ from existing literature currently available. A potential explanation could be the higher number of female (57%) compared to male (43%) respondents. Most probably, nowadays more women are interested in running than years ago. It is well known that the participation of female marathoners has increased in the last decades. For example, in the “Boston Marathon” from 1972 to 2017, female participation started at 2.8% in 1972 and reached 45.7% in 2016 (Knechtle et al., 2020). Similarly, in the “New York City Marathon” from 1970 to 2017, the number of both female and male finishers increased continuously across the years, where the increase was more pronounced in women. However, the number of female finishers never exceeded the number of male finishers (Vitti et al., 2020). For ultra-marathoners, differences were found between women and men regarding intensity and volume during training and their influence on race outcome. While volume of running kilometers during training per week was associated with race time in women, running speed during training was associated with race time in men (O’Loughlin et al., 2019). Regarding training, running speed during training sessions seems, however, more important for a successful race outcome than training volume. For both female and male recreational half-marathoners, running speed during training sessions, not training volume, was related to half-marathon race times (Friedrich et al., 2013). Future studies may investigate intensity in training for recreational runners.

### Limitations and Strength of the Study

Some limitations of our study should be noted. The survey is based on self-report, meaning that the reliability of the data depends on the conscientiousness of our subjects. However, we minimized this effect by using questions to control for race distance. Self-reports for this type of variable are valid if they are collected immediately or shortly after an event (Wilson et al., 2015). In this study, however, the average time between completion of the last event and completion of the questionnaire by the participants was not known (see inclusion criteria: self-reports refer to at least one running event completed within the past 2 years). Therefore, the validity of the self-report of the current study is unknown and not applicable. Therefore, the present investigation allows no conclusion regarding causality. However, it provides valuable information and indication of who is at the start of a running event, which is of interest especially for organizers of running events in general, but also for trainers, coaches and experts guiding athletes involved in running while adhering to some specific training and/or race distance. A strength of our study is the fact that we have a very large sample size regarding recreational endurance runners of different running distances.

## Conclusion

In summary, training volume increased with increasing race distance, male runners invested more time in training compared to female runners, marathon and ultra-marathon runners were more likely to seek advice from a professional trainer, and spring was most commonly reported across all subgroups as the planned season for racing.

## Data Availability Statement

The raw data supporting the conclusions of this article will be made available by the authors, without undue reservation.

## Ethics Statement

The study protocol is available online via https://springerplus.springeropen.com/articles/10.1186/s40064-016-2126-4 and was approved by the Ethics Board of St. Gallen, Switzerland on May 6, 2015 (EKSG 14/145). The patients/participants provided their written informed consent to participate in this study.

## Author Contributions

KW conceptualized, designed, and developed the study design and the questionnaires together with BK and CL. KW performed the data analysis. KW, DT, and BK drafted the manuscript. TR and VS helped in drafting the manuscript. BK and KW critically reviewed it. GW provided the technical support. All authors read and approved the final manuscript.

## Conflict of Interest

The authors declare that the research was conducted in the absence of any commercial or financial relationships that could be construed as a potential conflict of interest.
